# The C-Terminal Extension Unique to the Long Isoform of the Shelterin Component TIN2 Enhances Its Interaction with TRF2 in a Phosphorylation- and Dyskeratosis Congenita Cluster-Dependent Fashion

**DOI:** 10.1128/MCB.00025-18

**Published:** 2018-05-29

**Authors:** Nya D. Nelson, Lois M. Dodson, Laura Escudero, Ann T. Sukumar, Christopher L. Williams, Ivana Mihalek, Alessandro Baldan, Duncan M. Baird, Alison A. Bertuch

**Affiliations:** a Department of Molecular & Human Genetics, Baylor College of Medicine, Houston, Texas, USA; b Division of Hematology/Oncology, Department of Pediatrics, Baylor College of Medicine, Houston, Texas, USA; c Division of Cancer and Genetics, School of Medicine, Cardiff University, Cardiff, United Kingdom; d Bioinformatics Institute, Agency for Science Technology and Research, Singapore, Singapore

**Keywords:** TIN2, TPP1, TRF1, TRF2, casein kinase 2, dyskeratosis congenita, shelterin, telomere

## Abstract

TIN2 is central to the shelterin complex, linking the telomeric proteins TRF1 and TRF2 with TPP1/POT1. Mutations in *TINF2*, which encodes TIN2, that are found in dyskeratosis congenita (DC) result in very short telomeres and cluster in a region shared by the two TIN2 isoforms, TIN2S (short) and TIN2L (long). Here we show that TIN2L, but not TIN2S, is phosphorylated. TRF2 interacts more with TIN2L than TIN2S, and both the DC cluster and phosphorylation promote this enhanced interaction. The binding of TIN2L, but not TIN2S, is affected by TRF2-F120, which is also required for TRF2's interaction with end processing factors such as Apollo. Conversely, TRF1 interacts more with TIN2S than with TIN2L. A DC-associated mutation further reduces TIN2L-TRF1, but not TIN2S-TRF1, interaction. Cells overexpressing TIN2L or phosphomimetic TIN2L are permissive to telomere elongation, whereas cells overexpressing TIN2S or phosphodead TIN2L are not. Telomere lengths are unchanged in cell lines in which TIN2L expression has been eliminated by clustered regularly interspaced short palindromic repeat (CRISPR)/Cas9-mediated mutation. These results indicate that TIN2 isoforms are biochemically and functionally distinguishable and that shelterin composition could be fundamentally altered in patients with *TINF2* mutations.

## INTRODUCTION

The stability of the natural ends of linear chromosomes can be compromised by two major processes: progressive shortening with each round of DNA replication, due to the so-called end replication problem, and misrecognition of the ends as DNA double-strand breaks (DSBs), leading to activation of the DNA damage response (DDR) and DSB repair pathways. In most eukaryotes, telomeres, the specialized nucleoprotein structures at the chromosome termini, enforce chromosomal end stability through the activity of telomere-associated factors that inhibit activation of the DDR. These telomere-associated factors also play a crucial role in regulating the access and activity of the reverse transcriptase telomerase, which replenishes terminal telomeric repeats. In vertebrates, the shelterin complex, comprised of the TRF1, TRF2, RAP1, TIN2, TPP1, and POT1 proteins, plays an integral role in both of these functions (1–6).

**FIG 1:**
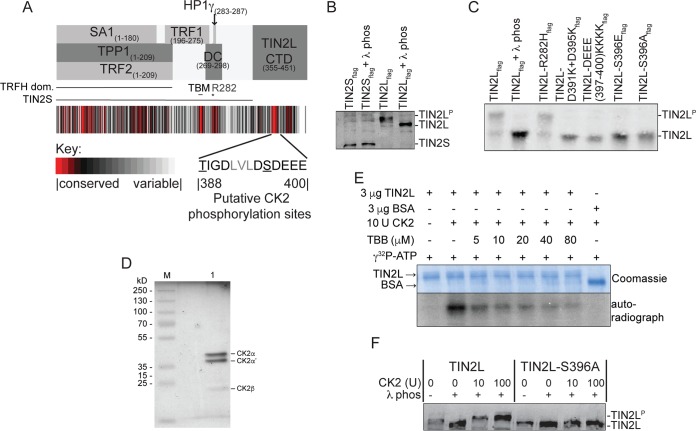
TIN2L phosphorylation is dependent upon an intact CK2 consensus site. (A) Evolutionary trace analysis and summary of known interaction regions in TIN2. The residues comprising the putative CK2 phosphorylation sites are displayed. The protein interaction domains, the region where all DC-causing mutations cluster (DC), the position of R282 (*), and the putative TBM, all of which are present in both the TIN2L and TIN2S isoforms, and the C-terminal domain unique to TIN2L (TIN2L CTD) are indicated. (B) Characterization of TIN2S and TIN2L phosphorylation. Flag-tagged TIN2S or TIN2L was analyzed by SDS-PAGE in the presence of Phos-tag reagent. Phosphorylated TIN2L (TIN2L^P^) is visible as a supershift compared to the λ phosphatase-treated TIN2L. (C) Characterization of TIN2L phosphorylation at S396 as in panel B. (D) Recombinant CK2 (NEB) analyzed by SDS-PAGE and silver staining. M, protein molecular mass marker; 1, CK2. (E) *In vitro* phosphorylation of TIN2L by CK2. Recombinant TIN2L purified from E. coli was incubated with CK2 and [γ-^32^P]ATP in the absence or presence of a CK2 inhibitor (TBB). (F) *In vitro* phosphorylation of partially purified TIN2L and TIN2L-S396A by CK2. Flag-tagged TIN2L or TIN2L-S396A from transiently transfected HEK 293T cells was dephosphorylated by incubation with λ phosphatase, purified via immunoprecipitation, and incubated with CK2. Phosphorylation status was assessed by SDS-PAGE in the presence of the Phos-tag reagent and compared to that of non-λ phosphatase-treated controls.

TIN2 is a central component of shelterin, linking the double-stranded telomeric binding proteins, TRF1 and TRF2, to the single-stranded telomeric binding protein POT1 via its interaction with TPP1 (7). Additionally, TIN2 interacts with the cohesin subunit SA1 (8). Whereas murine cells express a single TIN2 isoform, which is important for the protection of telomeres from DNA damage signaling and fusions via classical- and alternative nonhomologous end joining pathways (9), both a short (TIN2S) isoform and long (TIN2L) isoform of TIN2 are expressed in human cell lines (10) (Fig. 1A). TIN2L contains all 354 amino acids (aa) present in the shorter isoform along with an additional 97 aa at the C terminus via alternative splicing. Detailed interaction studies published to date have focused on residues shared between TIN2S and TIN2L or the full-length TIN2S isoform. For example, of the TIN2 crystal structures that have been reported, the first set consists of a small central peptide spanning aa 256 to 276, which includes a TRF homology (TRFH) domain binding motif (TBM), in complex with the TRF1- or TRF2-TRFH domains (9). The second set consists of the N-terminal domain of TIN2 (aa 2 to 202), which structurally resembles a TRFH domain, in a ternary complex with TPP1- and TRF2-TBMs (11). While the TIN2-TBM interacts with TRF2 with a much lower affinity than the TIN2-TRFH domain does, *in vivo* studies with TIN2S have demonstrated that residues within the TIN2-TBM can mediate a weak interaction with TRF2 when the interaction between the TIN2-TRFH and TRF2 is disrupted. Interestingly, in contrast to its interaction with the TRF2-TRFH domain, the TIN2-TBM interacts with a high affinity with the TRF1-TRFH domain and disruption of these residues in TIN2S has profound impact on TIN2S-TRF1 interactions *in vivo* (9). Whether the C-terminal TIN2L extension influences TIN2's interaction with its shelterin binding partners has not been determined. Similar to the case with the interaction studies, little is known regarding the functional contributions of TIN2L versus TIN2S at telomeres. Simultaneous loss of both TIN2 isoforms via knockdown has seemingly contradictory effects on telomerase regulation, due to destabilizing effects on TRF1, which is a negative regulator of telomere length (12), and decreased telomere association of TPP1, which is crucial for the recruitment of telomerase to the telomere (13).

Gene mutations associated with human disease often provide insight into previously unrecognized protein function, which may be true for TIN2. *TINF2*, the gene that encodes TIN2, is the second most commonly mutated gene in the telomere biology disorder dyskeratosis congenita (DC) (14, 15). DC is a complex syndrome characterized by bone marrow failure and cancer predisposition, pulmonary fibrosis, and a multitude of other clinical features. Underlying these medical problems are constitutionally very short telomeres (16). DC-associated *TINF2* mutations are most frequently *de novo*, yet, strikingly result in drastically short telomeres within a single generation (15). This is in contrast to autosomal dominant mutations in *TERT*, the catalytic component of telomerase, or *TERC*, the integral RNA, which are most often inherited and result in progressively shorter telomeres and increasing disease severity or multisystem involvement in successive generations (17, 18). The basis for this rapid telomere shortening remains to be fully elucidated. Notably, all *TINF2* mutations reported for patients with very short telomeres, whether missense, frameshift, or nonsense, map to a central 30-amino-acid region (residues 269 to 298; DC cluster) which is immediately C terminal to the TIN2-TBM and present in both TIN2S and TIN2L (8, 19–21) (Fig. 1A). While the most N-terminal truncation was shown to decrease TIN2S binding to TRF1, no universal effect of these mutations on TIN2S binding to TRF1, TRF2, or TPP1 has been observed (22, 23). Thus, it has been suggested that the impact of the TIN2 mutations could be on other interactions.

Consistent with this, TIN2 binds heterochromatin protein 1γ (HP1γ) via a binding motif within the DC cluster region (TIN2 residues 283 to 287) (24). HP1γ binds to H3 tails methylated at lysine 9 and, similar to TIN2 (8), is necessary for sister telomere cohesion (24). Some DC-associated mutations affect both HP1γ binding and sister telomere cohesion, leading to the proposal that DC-associated mutations cause decreased sister telomere cohesion, resulting in a loss of telomere lengthening via homologous recombination during embryogenesis. However, frameshift/nonsense mutations C terminal to this binding motif would not be expected to reduce HP1γ binding, as was observed for the Q298Rfs mutation. Notably, missense mutations have been found only from residues 280 to 291, while frameshift and nonsense mutations have been found throughout this region. These frameshift and nonsense mutations would obliterate any specific functions of the C-terminal region of TIN2S and TIN2L and could result in the expression of a truncated protein lacking the TIN2L C-terminal domain, as has been shown for two such mutations (22). Our identification of a young child with DC, very short telomeres, and an even more C-terminal K302Rfs mutation further raises the question of functions other than HP1γ binding contributing to the very short telomeres observed in these patients.

Additionally, it has been reported that while DC-associated *TINF2* mutations do not affect overall telomerase activity, they do result in decreased telomerase activity immunoprecipitated with TIN2S (25). However, a mouse model in which a DC-associated *TINF2* mutation results in decreased telomere length even in the absence of the telomerase RNA component (26) indicates that defects in telomerase recruitment alone are unlikely to account for the much more severe phenotype seen in patients with *TINF2* mutations.

Importantly, both TIN2L and TIN2S contain all known binding regions and the DC cluster (10). Functions that may be unique or specific to TIN2L at the telomere and any effects of DC-associated mutations on those functions remain unexamined. We hypothesized that TIN2L has roles at the telomere not shared with the shorter isoform and that those roles could be impacted upon by DC-associated mutations. In this study, we have identified differences in the ability of TIN2L and TIN2S to interact with TRF1 and TRF2 and a role for the DC cluster and phosphorylation specifically in TIN2L interactions. Additionally, we show that TIN2L and TIN2S overexpression have different effects on telomere length. These data suggest that TIN2S and TIN2L have differing roles within the shelterin complex and in telomere regulation and that the composition of the shelterin complex could be fundamentally altered in patients with DC-associated *TINF2* mutations.

## RESULTS

### TIN2L is phosphorylated by casein kinase 2 (CK2).

To examine whether TIN2L contains regions potentially important for functions that are not present in the more commonly studied shorter isoform (TINS), we first estimated conservation of residue types across mammalian orthologs of TIN2L (see Materials and Methods). The estimated degree of conservation is collated in Fig. 1A below the approximate or delineated TIN2 binding regions determined previously by crystallography, yeast two-hybrid, far-Western, and coimmunoprecipitation (co-IP) experiments (7, 8, 19, 20). While much of the TIN2 sequence is highly variable, we found that conserved regions are present and some coincide with the known interaction regions of TIN2. In particular, the region from residues 258 to 267, which contains the TBM and is sufficient for high-affinity TRF1 but not TRF2 interaction (9), consists almost entirely of residues that are identical across all mammalian sequences. A large fraction of the residues within the N-terminal TRFH domain are also highly evolutionarily constrained. Lastly, within the 30-aa region spanning residues 269 to 298, where all of the DC-associated TIN2 mutations have been reported to date (indicated as DC in Fig. 1A), only residues 280 to 291 showed signs of evolutionary constraint, coinciding with the HP1γ binding motif (residues 283 to 287). In addition to the evolutionary constraint in these regions shared by TIN2S and TIN2L, we identified two highly evolutionarily constrained regions within the C-terminal domain exclusive to TIN2L (TIN2L CTD) (Fig. 1A). The region consisting of residues 388 to 400 was the more highly constrained of the two.

Within this region are residues 396SDEE399, which conform to a CK2 recognition motif (S/T-X-X-E/D, where X represents a nonbasic residue) targeting phosphorylation of S396 (27, 28). The prediction for S396 phosphorylation and its phosphorylation by CK2 was robust across several protein phosphorylation prediction algorithms including NetPhos3.1 (29, 30), GPS3.0 (31), and PPSP (29). These residues were conserved across mammalian orthologs reported in the Uniprot database (see Fig. S1 in the supplemental material). Further supporting these data, endogenous TIN2L was previously found to be phosphorylated at S396 in human embryonic stem cells using high-throughput mass spectrometry (32). Unlike humans, mice express only one isoform of TIN2 (10). Mouse TIN2 includes a C-terminal region with high similarity to the C-terminal extension in human TIN2L, and the cognate residue to human S396, mouse S380, was also found to be phosphorylated in three separate mass spectrometry analyses (33–35).

CK2 is known to phosphorylate TRF1 (36). This phosphorylation of TRF1 is necessary for its stability, binding to telomeric DNA, and homodimerization, thereby contributing to the negative length regulation of telomeres. Because CK2 is important for the telomeric function of TRF1 and a putative CK2 phosphorylation site is present in TIN2L, we asked if TIN2L is phosphorylated and, if so, by CK2. To determine if TIN2L is phosphorylated, we utilized the reagent Phos-tag, which retards the migration of phosphorylated proteins through SDS-polyacrylamide gels (37). First, we analyzed transiently expressed, tagged proteins in HEK 293T cells and found that TIN2L migrated more rapidly through the gel following λ phosphatase treatment, indicating that the majority of TIN2L was phosphorylated (Fig. 1B). While TIN2S can be phosphorylated (38), the migration of TIN2S was unaffected by λ phosphatase treatment, indicating that this isoform is not predominantly phosphorylated in asynchronous cells (Fig. 1B) Charge swapping mutations in the CK2 recognition site (D391K+D395K and DEEE397-400KKKK) and mutation of S396 to either a phosphomimetic (S396E) or phosphodead (S396A) residue abolished TIN2L phosphorylation (Fig. 1C). The most common missense mutation in DC, R282H, did not affect TIN2L phosphorylation (Fig. 1C).

CK2 is an essential kinase. Inhibition of CK2, via either RNA interference or the CK2-selective chemical inhibitor 4,5,6,7-tetrabromo-2-azabenzimidazole (TBB), results in apoptosis, making it difficult to study phosphorylation by CK2 in an endogenous context (39). To overcome this and establish whether CK2 might be responsible for the observed TIN2L phosphorylation, we purified recombinant tagged TIN2L from Escherichia coli and determined the ability of recombinant CK2 purified from E. coli (New England BioLabs [NEB]) (Fig. 1D) to phosphorylate this TIN2L *in vitro*. TIN2L was phosphorylated by CK2 in the presence of [γ-^32^P]ATP, as indicated by the presence of radiolabeled TIN2L (Fig. 1E). This phosphorylation decreased in the presence of TBB, which drastically inhibits CK2 but not 33 other kinases (40), indicating that TIN2L phosphorylation was CK2 dependent. To determine if the residue phosphorylated by CK2 was S396, we overexpressed TIN2L or TIN2L-S396A in 293T cells, treated the cell lysates with λ phosphatase to remove any existing phosphorylations, partially purified TIN2 via immunoprecipitation, and then incubated it with recombinant CK2. The phosphorylation status was then determined using SDS-PAGE in the presence of Phos-tag, followed by Western blotting. Immunoprecipitated TIN2L not subjected to λ phosphatase treatment was prepared as a control. As shown in Fig. 1F, wild-type TIN2L was phosphorylated by CK2, while TIN2L-S396A was not. In summary, these data showed highly conserved residues corresponding to a CK2 recognition site in TIN2L, loss of phosphorylation of tagged expressed TIN2L upon mutation of the site in cells, and *in vitro* phosphorylation of wild-type, but not mutant, partially purified TIN2L by CK2 and which was reduced by a highly specific CK2 inhibitor. Combined with the previous mass spectrometry data indicating that S396 is phosphorylated in both mouse and human cells, these data strongly support the notion that TIN2L-S396 is phosphorylated by CK2 *in vivo*.

### The DC cluster and TIN2L phosphorylation enhance TIN2L's association with TRF2 *in vivo*.

**FIG 2:**
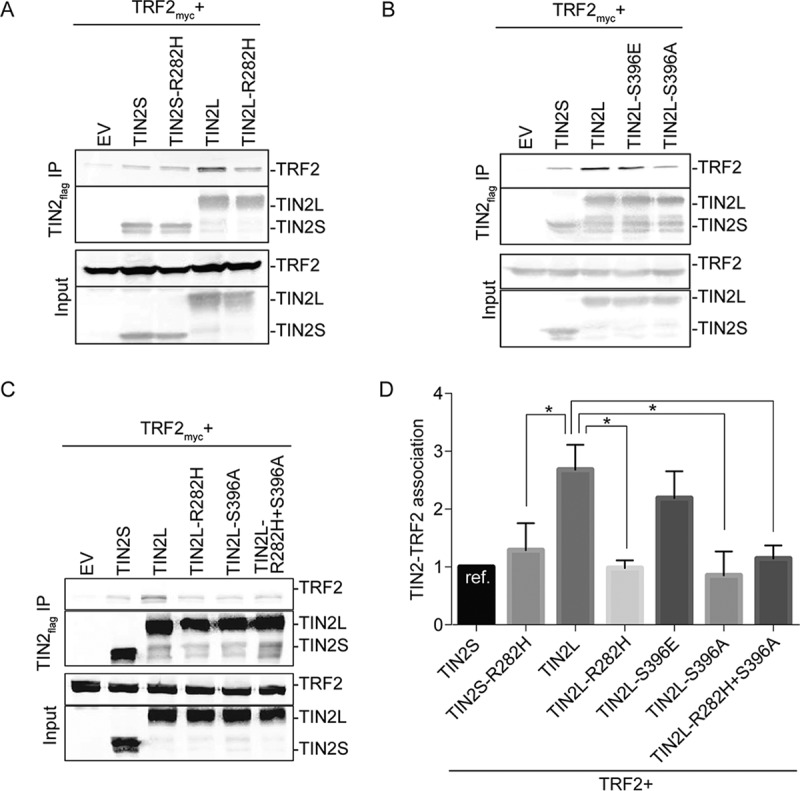
S396 phosphorylation and R282 cooperate to promote TIN2L binding to TRF2. (A) Representative co-IP of TRF2 with cotransfected wild-type TIN2S/L, TIN2S/L-R282H, or empty vector (EV). (B) Representative co-IP of TRF2 with cotransfected wild-type TIN2S/L, phosphomimetic TIN2L (TIN2L-S396E), phosphodead TIN2L (TIN2L-S396A), or EV. (C) Representative co-IP of TRF2 with cotransfected wild-type TIN2S/L, TIN2L-R282H, TIN2L-S396A, double mutant TIN2L-R282H+S396A, or EV. (D) Quantification of data in panels A, B, and C. For quantification, the amount of TRF2 coimmunoprecipitated was divided by the amount of TIN2 immunoprecipitated in order to account for any differences in TIN2 expression/pulldown. For each experiment, the value was then normalized to that of TIN2S. Error bars represent the SDs of several separate co-IP experiments. *, *P* < 0.008.

To determine the molecular effects of TIN2L phosphorylation, we examined the ability of transiently expressed epitope-tagged TIN2L to interact with the known TIN2S shelterin binding partners, which were also transiently expressed and epitope tagged, and the effect of mutation of the phosphorylation site on those interactions via coimmunoprecipitation. We also compared TIN2L interactions with that of TIN2S and the effect of the DC R282H mutation on TIN2L compared to TIN2S interactions. Interestingly, TIN2L interacted much more robustly with TRF2 than TIN2S (Fig. 2A; quantified in Fig. 2D). This is in contrast to what was previously published (10). However, as shown in Fig. 2D, this finding was very reproducible, with consistent results across multiple biological replicates. As previously reported, the most common missense mutation in TIN2 in DC patients, R282H, had no effect on TIN2S binding to TRF2 (Fig. 2A; quantified in Fig. 2D) (22, 23, 26). In contrast, the R282H mutation reduced TIN2L binding to TRF2 to levels similar to that of wild-type TIN2S, indicating an effect of the DC cluster that is manifest only within the context of the long isoform. Similarly, the phosphodead mutation greatly reduced TIN2L binding to TRF2, while the phosphomimetic mutation did not, indicating that TIN2L phosphorylation at S396 is critical for this enhanced interaction (Fig. 2B; quantified in Fig. 2D). The double mutant TIN2L-R282H+S396A did not decrease TRF2 binding beyond either mutation alone, indicating that the DC cluster and TIN2L phosphorylation site cooperate to enhance TRF2 binding to TIN2L (Fig. 2C; quantified in Fig. 2D).

**FIG 3:**
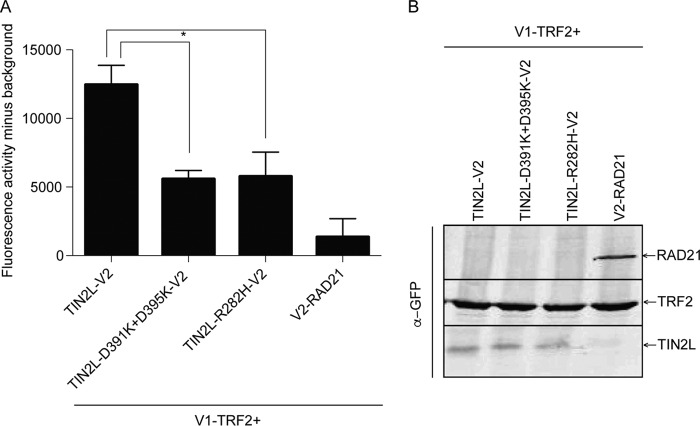
The protein complementation assay confirms the effects of the R282H mutation and a phosphorylation site mutation on TIN2L interaction with TRF2. (A) Quantification of fluorescence from coexpression of V1-TRF2 with TIN2L-V2, TIN2L-D391K+D395K-V2, or TIN2L-R282H-V2. Error bars represent the SDs from three separate transfections each measured in triplicate. *, *P* < 0.001. (B) Western blot exhibiting expression of the proteins assayed for panel A.

The importance of S396 phosphorylation and R282 in TIN2L-TRF2 interaction was also observed using the protein fragment complementation assay (PCA), with TIN2L and TRF2 fused with the C-terminal (indicated as V2) and N-terminal (indicated as V1) halves of the Venus yellow fluorescent protein variant, respectively. In this type of PCA, fluorescence is detected only when the proteins to which the split Venus halves are tagged come into close proximity, allowing reconstitution of N- and C-terminal fragments of the Venus yellow fluorescent protein (41). The fluorescence observed with cotransfection of TIN2L-V2 with V1-TRF2 was markedly reduced (Fig. 3) with TIN2L-R282H-V2 and TIN2L-D391K+D395K-V2, which abolishes TIN2L phosphorylation (Fig. 1C), consistent with a decreased interaction of these TIN2L mutants with TRF2. We found that the levels of expression of TIN2S and TIN2L differed greatly in these constructs, so the interaction between the TIN2 isoforms and TRF2 could not be compared using PCA.

**FIG 4:**
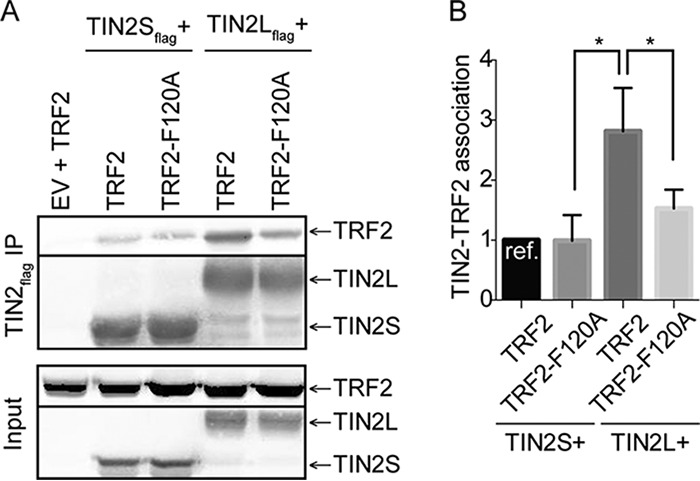
Interaction of TIN2L with TRF2 requires TRF2-F120. (A) Representative co-IP of TIN2S or TIN2L cotransfected with TRF2 or TRF2-F120A. (B) Quantification of data in panel A. Error bars represent the SDs from several separate co-IP experiments. For quantification, the amount of TRF2 coimmunoprecipitated was divided by the amount of TIN2 immunoprecipitated in order to account for any differences in TIN2 expression/pulldown. For each experiment, the value was then normalized to that of TIN2S. *, *P* < 0.05.

We next determined if the increased interaction of TRF2 with TIN2L involves the TRF2 TRFH domain. To do this, we employed a TRF2 TRFH domain mutation, F120A. TRF2-F120A was shown previously by transient transfection and coimmunoprecipitation to have no impact on TIN2S's interaction with TRF2, whereas mutation of the cognate residue in the TRF1 TRFH domain, F142A, drastically reduced TIN2S's interaction with TRF1. Nonetheless, the conformations of the TIN2-TBM bound to the TRFH domains of TRF1 and TRF2 are similar (9), and in one study, TRF2-F120A was shown to reduce transiently transfected TRF2's ability to interact with endogenous TIN2, although it was not determined if this was TIN2S or TIN2L (42). Reproducibly in our assays and in contrast to what was observed by transient transfection and coimmunoprecipitation for TRF2-F120A and TIN2S, we found that the TRF2-F120A mutation decreased TIN2L's binding to TRF2 (Fig. 4). This suggests that the C-terminal domain of TIN2L stabilizes the TIN2-TBM–TRF2-TRFH interaction *in vivo*.

### TIN2L binds less robustly than TIN2S to TRF1 but equivalently to TPP1.

**FIG 5:**
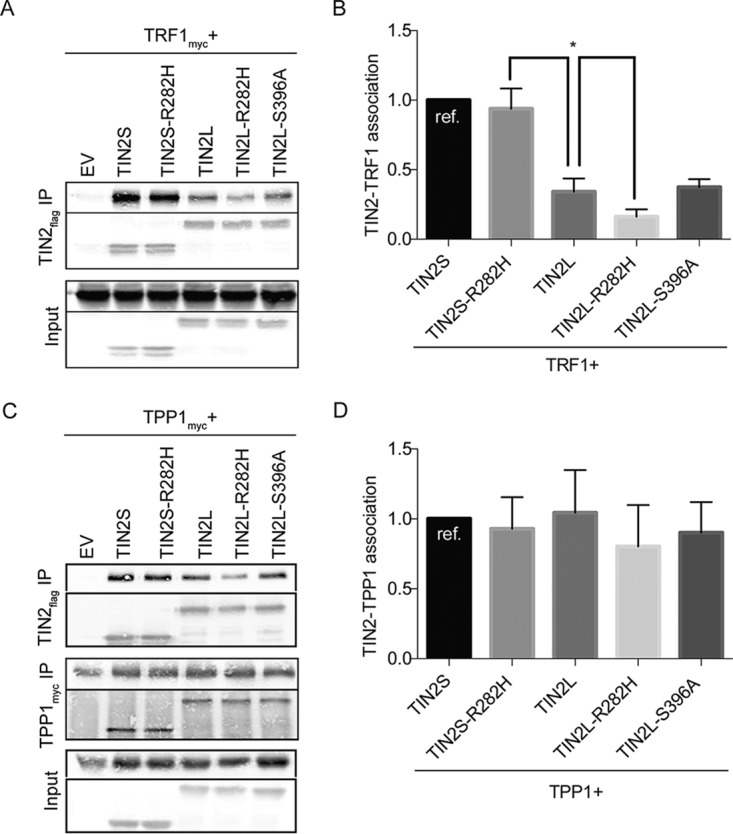
TIN2L interacts less robustly with TRF1 than TIN2S, but the two isoforms interact at similar levels with TPP1. (A) Representative co-IP of TRF1 cotransfected with wild-type TIN2S/L, TIN2S/L-R282H, TIN2L-S396A, or EV. (B) Quantification of data in panel A. For quantification, the amount of TRF1 coimmunoprecipitated was divided by the amount of TIN2 immunoprecipitated in order to account for any differences in TIN2 expression/pulldown. For each experiment, the value was then normalized to that of TIN2S. Error bars represent the SDs from several separate co-IP experiments. *, *P* < 0.02. (C) Representative co-IP of TPP1 cotransfected with wild-type TIN2S/L, TIN2S/L-R282H, TIN2L-S396A, or EV. (D) Quantification of data in panel C. For quantification, the amount of TPP1 coimmunoprecipitated was divided by the amount of TIN2 immunoprecipitated in order to account for any differences in TIN2 expression/pulldown. For each experiment, the value was then normalized to that of TIN2S. Error bars represent the SDs from several separate co-IP experiments.

We next examined how TRF1 interacted with TIN2L relative to TIN2S using transient expression and coimmunoprecipitation. Conversely and in striking contrast to TRF2, TRF1 interacted much more robustly with TIN2S than TIN2L (Fig. 5A; quantified in Fig. 5B). The R282H mutation had no effect on TIN2S binding to TRF1 but reduced TIN2L's ability to bind TRF1 even further. However, TIN2L phosphorylation appeared to play no role in interaction with TRF1 (Fig. 5A and B), unlike with TRF2 (Fig. 2B and D).

Lastly, having found that TRF2 interacted more robustly with TIN2L than TIN2S, and, conversely, TRF1 interacted more robustly with TIN2S than TIN2L, we next examined how the isoforms interacted with TIN2's third shelterin binding partner, TPP1. We found that in contrast to TRF1 and TRF2, the two isoforms interacted at similar levels with TPP1 in the coimmunoprecipitation assays (Fig. 5C; quantified in Fig. 5D).

**FIG 6:**
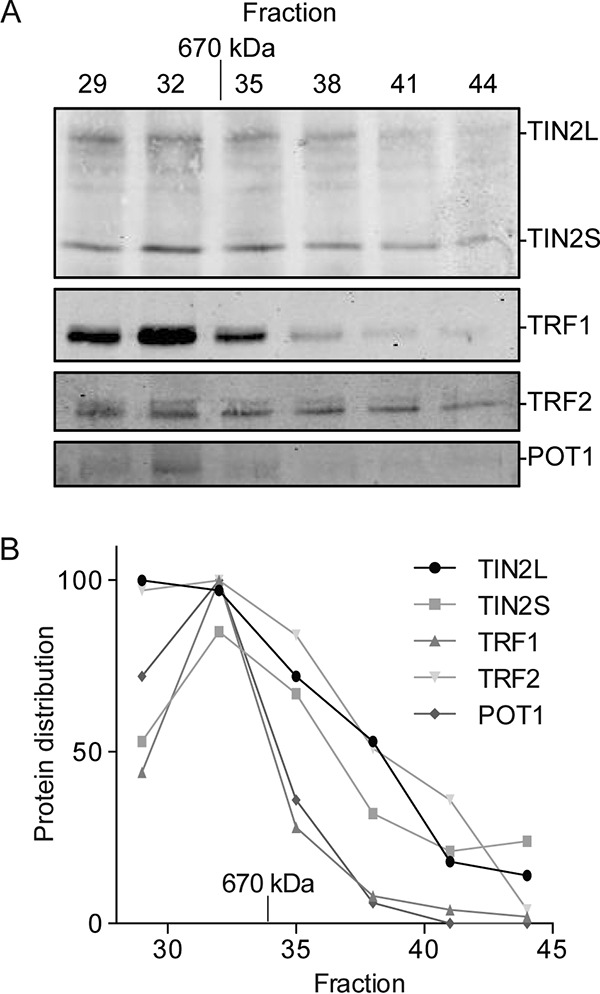
Endogenous TIN2L cofractionates with other shelterin components in HeLa cell nuclear extract. (A) Western blot analysis of shelterin components from size-based fractionation of HeLa cell nuclear lysate. HeLa cell nuclear extracts were subjected to gel filtration, and 0.5-ml fractions were taken beginning at a 30-ml elution volume. Aliquots of 50 μl were taken from the indicated fractions and analyzed for the presence of TIN2, TRF1, TRF2, and POT1 by immunoblotting. The molecular size indicated was determined using a molecular size standard. (B) Quantification of data in panel A. Values were normalized to the fraction with the largest amount of each respective protein.

To explore whether TIN2L is present in endogenous shelterin complexes, we subjected the nuclear fraction from a HeLa cell lysate to size-based fractionation under physiologic ionic strength using a gel filtration column. As previously reported, endogenous TIN2S, TRF1, TRF2, and POT1 cofractionated over a range of molecular masses larger than 670 kDa (Fig. 6) (7, 43, 44), which is larger than the size of a core shelterin complex consisting of TIN2S, a TRF2 homodimer, POT1, and TPP1 (306 kDa) (45), a TRF1 homodimer (115 kDa) (45), and 2 RAP1 molecules (expected size, 88 kDa). TIN2L was also present in these fractions, consistent with it being in a complex with the shelterin components. Given the larger-than-expected cumulative size of the proteins that cofractionated, TIN2S and TIN2L could simultaneously be present within a single shelterin complex.

### TIN2L is not required for viability or normal telomere length maintenance in transformed cell lines.

**FIG 7:**
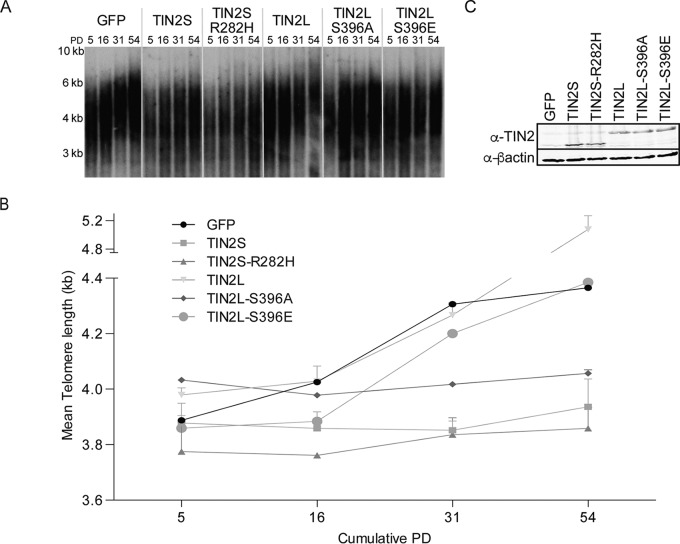
Loss of TIN2L phosphorylation inhibits progressive telomere elongation in telomerase-positive HT1080 cells. (A) Representative telomere Southern blot analysis of telomere length in kilobase pairs (kb) over time in HT1080 cells overexpressing TIN2S, TIN2S-R282H, TIN2L, TIN2L-S396A, TIN2L-S396E, or GFP control. HT1080 cells overexpressing the indicated TIN2 proteins were collected at various times following induction with lentivirus and blastocidin selection and analyzed by the terminal restriction fragment assay. (B) Quantification of data in panel A using densitometry analysis. Error bars indicate the SDs from two separate terminal restriction fragment assays. (C) Western blot showing TIN2 expression levels in each cell line. Total cell protein lysates were prepared using 2× Laemmli buffer.

Given the differences in shelterin component binding between TIN2L and TIN2S, we next sought to determine if they might also differentially impact telomere length regulation. The role of TIN2L S396 phosphorylation in telomere length regulation was of particular interest given CK2's role in telomere length regulation via its phosphorylation of TRF1 (43). First, we stably overexpressed TIN2S, TIN2S-R282H, TIN2L, TIN2L-S396A, TIN2L-S396E, or a green fluorescent protein (GFP) control in the HT1080 cell line and determined the telomere length by Southern blotting over successive population doublings (Fig. 7). We found that the telomeres progressively elongated in the HT1080 GFP control cells. Telomere elongation in this control cell line has been observed by others (19, 21, 25, 26, 46) and may reflect resetting of telomere length in sublines that had previously undergone stochastic shortening. While telomeres progressively elongated in the GFP control cell lines, overexpression of either TIN2S or TIN2S-R282H inhibited this progressive elongation, consistent with previous reports demonstrating impairment of telomere maintenance upon TIN2S overexpression (24, 25). In contrast, telomeres continued to progressively elongate in cells overexpressing wild-type TIN2L, which was a consistent observation in separately generated cell lines (Fig. 7; see also Fig. S2). This result could indicate that TIN2L simply lacks activity (as with GFP) or specifically lacks the inhibitory activity of TIN2S. However, whereas telomeres also progressively elongated in cells overexpressing TIN2L with the phosphomimetic S396E mutation, they did not in cells overexpressing TIN2L with the phosphodead S396A mutation (Fig. 7; see also Fig. S3). The differences in telomere length changes could not be attributed to differences in levels of overexpression, which were equivalent in each of the cell lines (Fig. 7C; see also Fig. S2 and S3), or to differences in population doubling times or cumulative population doublings for the cell lines, which were equivalent (Fig. S4). Taken together, these results are most consistent with the wild-type TIN2L lacking inhibitory activity and the S396A mutation resulting in TIN2L becoming TIN2S-like with respect to telomere length regulation, similar to the effect it has on TIN2L's TRF2 binding (Fig. 2B and D).

To explore the role of endogenous TIN2L in telomere length regulation, we used the clustered regularly interspaced short palindromic repeat (CRISPR)/Cas9 system (47) to modify the genomic *TINF2* locus. We designed guide RNAs that targeted TIN2L-specific exons 7 and 8 for mutagenesis (Fig. S5A and B), thereby creating cell lines that still encoded TIN2S but either no longer encoded TIN2L protein or encoded a protein that was truncated N terminal to S396. We characterized four unique compound heterozygous cell lines, three in the HEK 293T-derived Flp-In T-REx cell line and one in the HT1080 cell line (Fig. S5C). Clonal lines that were isolated but found to not contain mutations in *TINF2* served as controls. The Flp-In T-REx lines 285-F3 and 285-F10 had frameshift mutations in exon 7 of each allele, whereas the cell line 286-R had frameshift mutations in exon 8, N terminal to S396. The HT1080 lines 286-6A, 286-6B, and 286-6C, which were derived from the same clone, had an in-frame deletion of 15 bp, encoding a protein lacking amino acids 387 to 392, and a frameshift mutation encoding a protein truncated at amino acid 394.

**FIG 8:**
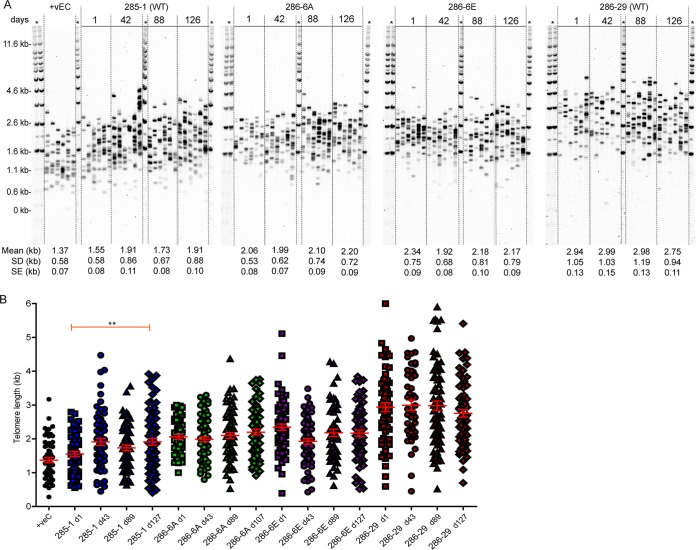
Telomere lengths of TIN2L mutant HT1080 cell lines are indistinguishable from TIN2L wild-type lines. (A) XpYp STELA of DNA isolated at the indicated numbers of days from the point of clonal line derivation. Mean lengths, SDs, and SEs are indicated. +vEC, 293 cell line. (B) Individual telomere lengths in the designated lines at the designated days.

The TIN2L-deficient clones were viable, although some clones exhibited decreased growth compared to that of the wild type (Fig. S6). Single telomere length analysis (STELA), which provides a sensitive measure of changes in telomere length, revealed that the average 17p or XpYp telomere length in independently derived clones varied within the range observed in clones with intact TIN2L and over successive population doublings (Fig. 8; see also Fig. S7). These results suggest that in these telomerase-positive cancer (HT1080) and transformed (Flp-In T-REx) cell lines, TIN2L is not required for viability or normal telomere length maintenance.

**FIG 9:**
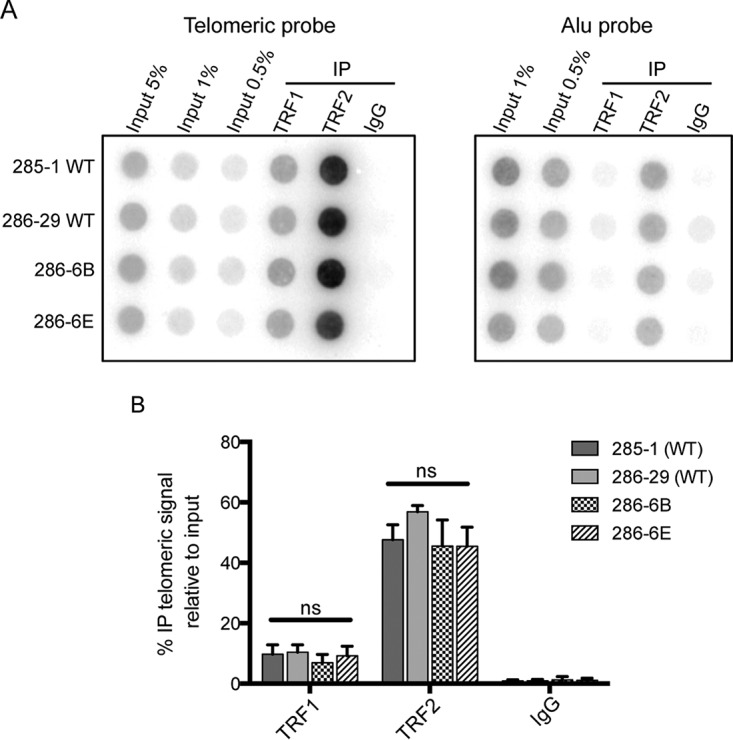
The telomere association of TRF1 and TRF2 is unaffected by loss of TIN2L function. (A) Endogenous TRF1 and TRF2 were immunoprecipitated from lysates prepared from formaldehyde-cross-linked cells. Association with telomeric DNA was assessed by Southern blotting. IgG and Alu probes were included as controls for nonspecific association. (B) Average percent telomeric DNA in IP relative to input, ±1 SD (*n* = 3).

In contrast to our approach, Kim et al. studied the effect of CRISPR/Cas9 knockout of both TIN2 isoforms in HeLa cells (48). They found that loss of both TIN2 isoforms results in a reduction in TRF2 but not TRF1 association with telomeres in chromatin immunoprecipitation (ChIP) assays. Therefore, we determined the effect of loss of TIN2L protein alone on the association of TRF2 with telomeres. Although TIN2L interacted preferentially with TRF2 in co-IP experiments (Fig. 2), the absence of intact TIN2L protein had no impact on the telomere association of TRF2 (Fig. 9). The interaction of TRF1 with telomeres was similarly unaffected. Together with the telomere length data, these results suggest that TIN2L and TIN2S have redundant functions in these cell lines.

## DISCUSSION

Prior studies have focused on the shorter isoform of TIN2; however, our data reveal differences in the interactions of TIN2S and TIN2L with TRF1 and TRF2, suggesting that the composition of the shelterin complex and the interactions within may be even more complex than previously thought (7, 43, 45, 49). Although previous studies have indicated that DC-associated mutations do not uniformly impact TIN2S interaction with TRF1, TRF2, and TPP1 (22, 23), the impact of the most common DC-associated mutation on TIN2L's ability to bind to both TRF1 and TRF2 leaves open the possibility that the composition of the shelterin complex is fundamentally altered in patients with *TINF2* mutations, potentially contributing to their devastatingly short telomeres.

This study demonstrated that the increased interaction between TIN2L and TRF2, in comparison to TIN2S, requires the residue most commonly mutated in DC, R282, as well as phosphorylation of S396, and that R282 and phospho-S396 cooperate or redundantly promote this enhanced interaction (Fig. 2 and 3). These findings were unexpected, as the primary TRF2 binding region in TIN2S resides within its N-terminal TRFH domain (11, 20) and the most common mutation in DC has no effect on TIN2S-TRF2 interaction (22, 23, 26). Additionally, we found that the TRF2-TRFH domain is required for this increased interaction, which was similarly unexpected based on the lack of impact of the TRF2-F120A mutation on TIN2S-TRF2 interaction *in vivo* (9). How the TIN2L-specific C-terminal domain influences TRF2 binding remains to be determined. As this region is predicted to be intrinsically disordered, structural studies will likely prove challenging.

While overexpression of TIN2S inhibited progressive telomere elongation in HT1080 cells, overexpression of TIN2L did not (Fig. 7; see also Fig. S3 in the supplemental material). It is interesting to consider the possible mechanisms for this difference in light of protein-protein interaction data. It has previously been shown that TIN2S prevents inactivation of TRF1 via inhibition of poly-ADP ribosylation of TRF1 by tankyrase (12). TRF1 is a negative regulator of telomere length (46). Since TRF1 interacts much more robustly with TIN2S than TIN2L (Fig. 5A and B), the discrepancy in telomere length maintenance could be due to effects on TRF1. Overexpression of TIN2L with a phosphodead mutation also inhibited progressive telomere elongation (Fig. 2; see also Fig. S3). If the failure of telomeres to elongate in cells overexpressing TIN2S is indeed due to TRF1 stabilization, this is unlikely to be the mechanism for TIN2L-S396A-mediated failure of telomere elongation, since wild-type TIN2L and TIN2L-S396A interacted with TRF1 at similar levels (Fig. 5A and B). It is therefore possible that phosphorylated TIN2L has a role in telomere maintenance unique from either TIN2S or unphosphorylated TIN2L.

When endogenous full-length TIN2L was eliminated using the CRISPR/Cas9 system, no consistent effect on telomere length was observed, which was surprising given the highly conserved CK2 consensus site within this region. While it is possible that TIN2S and TIN2L have redundant functions in telomere length maintenance, it is equally possible that TIN2L has other roles in telomere function. For example, we found that TIN2L interacts with TRF2 via F120 (Fig. 4), a residue within its TRFH domain that is known to be critical for TRF2 binding to Apollo and SLX4 (9, 50). We speculate that TIN2L may compete with these or other proteins that bind TRF2 via the TRFH domain. Therefore, in patients with the R282H mutation, the reduced interaction of TIN2L with TRF2 could result in a reduction in TIN2L binding to TRF2 at the telomere, thereby allowing increased recruitment of Apollo (51), SLX4 (50), or other factors that may drive telomere shortening. Future studies will address this hypothesis.

Due to the difficulties in exploring the interactions of each isoform and multiple mutations with TRF1, TRF2, and TPP1 in an endogenous setting, these results were obtained using a transient-transfection system with epitope-tagged proteins expressed at higher than endogenous levels. The use of epitope tagging of TIN2S and TIN2L was necessary, as any antibody designed to bind TIN2S would also, by necessity, bind to TIN2L. This may raise concern that the differential interactions are directly or indirectly the result of the overexpression. However, using the same set of constructs expressed at the same level under identical conditions in both co-IP and PCA, we observed isoform- and allele-specific effects that suggest targeted impact on protein interaction. TIN2S and TIN2L interacted similarly with TPP1, and the interaction was not impacted by either R282H or S396A. Yet we observed very specific results with TIN2S and TIN2L interactions with TRF1 and TRF2 and very specific effects of R282H or S396A on TIN2S and TIN2L interactions. This suggests that the differential interactions observed were unlikely to be due to nonspecific effects such as aggregation.

This study indicates that the two TIN2 isoforms preferentially interact with different members of the shelterin complex. We therefore anticipate that they could play different roles in telomere regulation. These data indicate that the most common DC-associated *TINF2* mutation greatly affects the ability of TIN2L, but not TIN2S, to interact with members of the shelterin complex. While the field has largely overlooked the longer isoform of TIN2, future studies will need to take both isoforms into account.

## MATERIALS AND METHODS

### Comparative analysis of protein sequences.

We used a real-value evolutionary trace method (52) to assign a score to the degree of conservation of protein residues from TIN2 orthologs reported by the Ensembl database (53). A BLAST search against the NCBI's RefSeq (54) database, using the TIN2L sequence as the query, was performed to confirm results from the Ensembl database. We chose to base our analysis on mammalian sequences only, as Ensembl did not report any TIN2 orthologs in vertebrates other than mammals and amphibians, and the BLAST search returned only mammalian sequences with matches to the last three exons of the TIN2 gene.

### Prediction of TIN2 phosphorylation and kinase-specific predictions.

Human TIN2L sequence (Uniprot identifier Q9BSI4.1) was analyzed using NetPhos3.1 (29, 30), GPS3.0 (31), and PPSP (29) algorithms via their respective servers (http://gps.biocuckoo.org, http://www.cbs.dtu.dk/services/NetPhos/, and http://ppsp.biocuckoo.org/, each accessed 18 November 2017).

### Vectors and mutagenesis.

The pcDNA3.1-flag-2×HA-TIN2S and pcDNA-flag-2×HA-TIN2S-R282H vectors have been previously described (22). The cDNA encoding the C terminus of TIN2L was amplified from MGC-12628 (ATCC) and subcloned 3′ to the penultimate codon in pcDNA3.1-flag-2×HA-TIN2S to generate pcDNA3.1-flag-2×HA-TIN2L. pcDNA3.1-flag-2×HA-TIN2L-R282H, pcDNA3.1-flag-2×HA-TIN2L-D391K+D395K, and pcDNA3.1-flag-2×HA-TIN2L-DEEE(397-400)KKKK were generated using site-directed mutagenesis as previously described (22). pLenti6.3-GFP, pLenti6.3-TIN2S, pLenti6.3-TIN2L, pLenti6.3-TIN2L-S396A, and pLenti6.3-TIN2L-S396E were generously provided by Kenneth Scott (Baylor College of Medicine). The C termini of TIN2L-S396E and TIN2L-S396A were amplified from their respective pLenti6.3 vectors and subcloned 3′ to the penultimate codon in pcDNA3.1-flag-2×HA-TIN2S to generate pcDNA3.1-flag-2×HA-TIN2L-S396E and pcDNA3.1-flag-2×HA-TIN2L-S396A. pLenti6.3-TIN2S-R282H was generated from pcDNA3.1-flag-2×HA-TIN2S-R282H via subcloning. pcDNA3.1-flag-2×HA-TIN2L-R282H+S396A was generated via subcloning from plasmids containing the respective mutations. myc-TPP1 was amplified from pLpcx-myc-TPP1 (generously provided by Susan Smith, New York University [NYU]) and subcloned into pcDNA3.1 to generate pcDNA3.1-myc-TPP1. TIN2L was amplified from pcDNA3.1-flag-2×HA-TIN2L and subcloned into pET28SUMO (generously provided by Ming Lei, University of Michigan) to generate pET28SUMO-TIN2L. TIN2L, TIN2L-D391K+D395K, and TIN2L-R282H were amplified from their respective pcDNA3.1 vectors and subcloned into GCN4 leucine zipper-Venus 2 (C-terminal half; V2) to generate TIN2L-V2, TIN2L-D391K-V2, and TIN2L-R282H-V2. V1-TRF2 and V2-RAD21 have been previously described (2). pcDNA3.1-myc-TRF1, pcDNA3.1-myc-TRF2, pcDNA3.1-myc-TRF2-F120A, and pSP73Sty11 were all a generous gift from Titia de Lange (Rockefeller University).

### Immunoblotting.

Cells were resuspended in ice-cold lysis buffer (50 mM Tris-HCl at pH 7.5, 1 mM EDTA, 400 mM NaCl, 1% Triton X-100, 0.1% SDS, 1 mM dithiothreitol [DTT], 1 mM phenylmethylsulfonyl fluoride [PMSF], and 1× protease inhibitor cocktail III [Calbiochem]) and incubated for 10 min on ice prior to addition of an equal amount of ice-cold water. The lysates were then centrifuged at 4°C and 20,800 × *g* for 10 min, and the pellet was discarded. Protein concentration was determined using the bicinchoninic acid (BCA) protein assay kit (Pierce). Lysates were resolved on 10% SDS-polyacrylamide gels and transferred to nitrocellulose membranes. The membranes were probed with at least one of these primary antibodies: rabbit polyclonal anti-FLAG (Sigma-Aldrich), rabbit polyclonal anti-c-Myc (Sigma-Aldrich), rabbit polyclonal anti-TIN2 number 865 (kindly provided by Titia de Lange, Rockefeller University), mouse monoclonal anti-β-actin (Sigma-Aldrich), rabbit polyclonal anti-TRF1 (Santa Cruz), rabbit polyclonal anti-TRF2 (Santa Cruz), rabbit polyclonal anti-POT1 (Abcam), and rabbit polyclonal anti-GFP (Abcam). The appropriate IRDye 800CQ-conjugated secondary antibody (Li-Cor) was then used, and blots were visualized using the Li-Cor Odyssey infrared imaging system. Blots were stripped by incubation in 0.1 M NaOH for 10 min at room temperature and reprobed. Immunoblots were quantified using Odyssey v3.0 software (Li-Cor).

### Phosphate affinity SDS-PAGE using Phos-tag.

Lysates were prepared as described above for immunoblotting, with the addition of 1× phosphatase inhibitor cocktail II (Sigma-Aldrich) to the lysis buffer to prevent dephosphorylation. As a control, protein extracts were also prepared in the absence of phosphatase inhibitors and treated with 400 U of λ phosphatase (NEB) for 30 min at 30°C. Lysates were resolved on a 10% (wt/vol) acrylamide gel with 100 μM Phos-tag (Wako) prepared according to the manufacturer's instructions. Prior to transfer, the gel was soaked in transfer buffer with 1 mM EDTA for 10 min at room temperature with shaking. Subsequent steps were carried out as described above for immunoblotting.

### TIN2L protein purification.

Protein purification was conducted similarly to the method previously described for TIN2S, with slight modifications (9). Human TIN2L in a modified pET28b vector with a SUMO site between the 6×His tag and the N terminus of TIN2L was expressed in E. coli BL21(DE3). Following induction with 0.1 mM isopropyl-β-d-thiogalactopyranoside (IPTG), cells were grown for 24 h at 16°C and then harvested by centrifugation. Cells were then resuspended in lysis buffer (50 mM phosphate buffer at pH 7.2, 0.5 mM 2-mercaptoethanol, 10% glycerol, 1 mM PMSF, 400 mM NaCl, 3 mM imidazole, 0.1 mg/ml of lysozyme, 1× protease inhibitor cocktail set III [Calbiochem]) and lysed via sonication. The lysate was then cleared via centrifugation and incubated overnight at 4°C with nickel-nitrilotriacetic acid (Ni-NTA)–agarose beads (Qiagen). The bead-lysate slurry was applied to a column and washed with 10 column volumes of wash buffer (50 mM phosphate buffer at pH 7.2, 0.5 mM β-mercaptoethanol, 10% glycerol, 1 mM PMSF, 400 mM NaCl, 20 mM imidazole) prior to addition of 1.5 column volumes of elution buffer (50 mM phosphate buffer at pH 7.2, 0.5 mM β-mercaptoethanol, 10% glycerol, 1 mM PMSF, 400 mM NaCl, 250 mM imidazole, 1× protease inhibitor cocktail set III [Calbiochem]). The eluate was then concentrated using an Amicon Ultra 10K centrifugal filter (Millipore) prior to separation on a HiLoad 16/600 Superdex 200 pg (GE Healthcare Life Sciences) gel filtration column equilibrated with gel filtration buffer (25 mM Tris at pH 8.0, 150 mM NaCl, 5 mM DTT). The fractions containing TIN2L were pooled, concentrated using an Amicon Ultra 10K centrifugal filter (Millipore), and stored at −80°C until use.

### *In vitro* phosphorylation.

Three micrograms of recombinant TIN2L purified from E. coli was incubated with 10 U of CK2 (NEB) and 10 μCi of [γ-^32^P]ATP in 1× CK2 reaction buffer (NEB) at 30°C for 30 min. As a control, 3 μg of bovine serum albumin (BSA; NEB) was incubated with CK2 under the same conditions. To confirm that the observed phosphorylation was carried out by CK2, the reaction was also performed in the presence of increasing concentrations of 4,5,6,7-tetrabromo-2-azabenzimidazole (TBB), a CK2 inhibitor. Following the phosphorylation reaction, proteins were resolved on a 10% SDS-PAGE gel, which was then exposed to a PhosphorImager screen. *In vitro* phosphorylation assays were also performed using flag-tagged TIN2L or TIN2L-S396A partially purified from 293T cells. Twenty-four hours after transfection with 5 μg of DNA using Lipofectamine and Plus Reagent (Invitrogen) according the manufacturer's instructions, cells were lysed as described above. Phosphatase inhibitor cocktail II (Sigma-Aldrich) was added to one-quarter of the lysate, which was set aside as a control. The remainder of the lysate was treated with 400 U of λ phosphatase (NEB) for 30 min at 30°C. The phosphatase was then inactivated by the addition of 50 mM EDTA and 1× phosphatase inhibitor cocktail II (Sigma-Aldrich). The lysates, including the reserved control, were then incubated overnight at 4°C with mouse monoclonal anti-FLAG M2 magnetic beads (Sigma-Aldrich) to isolate flag-tagged TIN2L or TIN2L-S396A. Beads were washed four times with a 1:1 dilution of lysis buffer and then resuspended in 1× CK2 buffer (NEB) supplemented with 200 μM ATP, 1× phosphatase inhibitor cocktail II (Sigma-Aldrich), 1× protease inhibitor cocktail III (Calbiochem), and the desired amount of CK2. Phosphorylation was carried out at 30°C for 30 min prior to analysis using phosphate affinity SDS-PAGE with Phos-tag.

### Coimmunoprecipitation.

Coimmunoprecipitations were conducted similarly to the method previously described (7). For TIN2 coimmunoprecipitations with TRF1, TRF2, or TPP1, 3 × 10^6^ HEK 293T cells were cotransfected with 5 μg of each plasmid using the Lipofectamine and Plus reagent (Invitrogen) according to the manufacturer's instructions. Twenty-four hours after transfection, the cells were lysed as described above for immunoblotting. Half a percent of the supernatant was reserved as input. Supernatants were incubated overnight at 4°C with 60 μl of mouse monoclonal anti-FLAG M2 magnetic beads (Sigma-Aldrich) or 2 μg of mouse monoclonal anti-Myc 9E10 (Abcam). For myc pulldowns, 60 μl of protein G Plus-agarose beads (Calbiochem) was added during the final hour. Beads were washed four times with a 1:1 dilution of lysis buffer prior to elution with Laemmli loading buffer. Proteins were analyzed by immunoblotting. Western blots were quantified using Odyssey V3.0 (Li-Cor).

### PCA.

PCA was carried out as previously described (2).

### Stable overexpression cell lines.

HT1080 cells were infected with pLenti6.3-GFP, pLenti6.3-TIN2S, pLenti6.3-TIN2S-R282H, pLenti6.3-TIN2L, pLenti6.3-TIN2L-S396A, or pLenti6.3-TIN2L-S396E lentivirus produced in HEK 293T cells. HT1080 cells overexpressing the genes of interest were then selected by incubation with selection medium containing blasticidin. Beginning 2 weeks after initial viral induction (time zero), cells were counted and plated every 3 to 4 days to follow growth and population doublings, and cells pellets were saved at −80°C for further analysis.

### Measurement of telomere length.

Measurement of bulk telomere terminal restriction fragment length was determined by Southern blotting as previously described (55), with the following specifications. Genomic DNA was isolated using the DNeasy blood and tissue kit (Qiagen) and subjected to digestion with HinfI, RsaI, and RNase A (NEB). Digested DNA was separated on a 1% agarose gel via pulsed-field gel electrophoresis and then transferred to a Zetaprobe GT membrane (Bio-Rad) for detection of telomeric sequence by hybridization with an 800-bp telomeric probe derived from a pSP73Sty11 fragment labeled with [α-^32^P]dCTP using Klenow fragment (46). Telomere length was determined using ImageQuant software (GE Healthcare Life Sciences) and Telorun (http://www4.utsouthwestern.edu/cellbio/shay-wright/research/sw_lab_methods.htm).

Single telomere length analysis (STELA) at 17p and XpYp was performed as previously described (56).

### CRISPR/Cas9 cell line creation.

Guide RNAs (gRNAs) targeting exons 7 and 8 of the *TINF2* locus were designed using the CRISPR Design tool (http://crispr.mit.edu/) (57). Three guides with quality scores of 76 or greater were chosen. Cleavage efficiency of the gRNAs was determined using the Guide-it mutation detection kit (TaKaRa) according to the manufacturer's instructions. HT1080 and Flp-In T-REx cell lines were transfected with 5 μg of pGS-gRNA-Cas9-Puro (Genscript) containing the desired gRNA using Lipofectamine and Plus (Invitrogen) according to the manufacturer's instructions. Two days after transfection, the host cell line was diluted and plated to form colonies. After expansion of the clones, 48 colonies from each gRNA were screened for mutations in the *TINF2* gene initially by sequencing PCR products amplified from the surrounding genomic region. Those with products with abnormal sequences were TopoTA cloned and 10 TopoTA clones sequenced to determine the sequences on each allele. Cells from colonies of interest were counted and plated every 3 to 4 days to follow growth and population doublings, and cell pellets were saved at −80°C for further analysis.

### Isolation of endogenous nuclear complexes.

HeLa cell nuclear complexes were examined as previously described (43). Briefly, nuclei from 7 × 10^9^ HeLa cells were extracted using 0.5 M KCl (58). Extracts were dialyzed into S-300 buffer (50 mM Tris at pH 7.5, 150 mM KCl, 0.2 mM EDTA, 0.025% NP-40, 0.5 M dithiothreitol, 1× cOmplete protease inhibitor [Roche]) and clarified by centrifugation. The dialyzed sample was concentrated using an Amicon Ultra 10K centrifugal filter (Millipore) prior to fractionation on a HiLoad 16/600 Superdex 200 pg (GE Healthcare Life Sciences) gel filtration column equilibrated with S-300 buffer. Half-milliliter fractions were taken beginning at a 30-ml elution volume and analyzed by immunoblotting.

### Telomere ChIP.

Cells were fixed in 1% formaldehyde for 30 min at room temperature, followed by lysis in radioimmunoprecipitation (RIPA) buffer (50 mM Tris-HCl at pH 8, 5 mM EDTA, 150 mM NaCl, 0.5% sodium deoxycholate, 1% NP-40, 0.1% SDS, 1 mM PMSF, and 1× protease inhibitor cocktail III [Calbiochem]). The lysates were sonicated in a Diagenode Bioruptor for 10 min, 3 times at the high setting, to generate ∼1-kb DNA fragments. Cellular debris was pelleted by centrifugation at 4°C and 20,000 × *g* for 10 min, and the protein concentration was assessed using the BCA protein assay kit (Pierce). For immunoprecipitation, 600 μg of the lysate was incubated with the corresponding antibodies (3 μg) overnight at 4°C: rabbit anti-TRF2 (Novus; NB110-57130), rabbit anti-TRF1 (Abcam; ab1423), and rabbit IgG (Santa Cruz). The next day, 45 μl of protein G magnetic beads (Pierce) was added, and after 2 h, the beads were washed 2 times in RIPA buffer, 4 times in wash buffer (100 mM Tris-HCl at pH 8.5, 500 mM LiCl, 1% NP-40, 1% sodium deoxycholate) and another 2 times in RIPA buffer. Following two washes in 1× Tris-EDTA (TE), proteins were eluted in 1× TE–1% SDS and incubated in 65°C overnight to reverse the cross-links. The samples were then treated with RNase A (20 μg) and proteinase K (40 μg) and subjected to phenol-chloroform extraction. After ethanol precipitation, dot blotting was performed on a Zeta-probe membrane (Bio-Rad) to hybridize the DNA with an 800-bp radiolabeled TTAGGG probe to assess the amount of immunoprecipitated telomeric DNA. An oligonucleotide Alu probe labeled at the 5′ end with α-^32^P was used as a negative control. ImageQuant software was used to quantify the signal intensity of telomeric or Alu IP relative to the corresponding input signals.

### Cell line authentication.

Cell lines were authenticated by short tandem repeat DNA analysis at the M. D. Anderson Cancer Center Characterized Cell Line Core Facility (1 September 2017).

## Supplementary Material

Supplemental material
