# Supplementary material for: The C-Terminal Extension Unique to the Long Isoform of the Shelterin Component TIN2 Enhances Its Interaction with TRF2 in a Phosphorylation- and Dyskeratosis Congenita Cluster-Dependent Fashion
Source: Mol Cell Biol. 2018 May 29;38(12):e00025-18. doi: 10.1128/MCB.00025-18 (PMC5974431; doi:10.1128/MCB.00025-18)
Supplement: Supplemental material [file supp_38_12_e00025-18__index.html]

Supplemental material 

# The C-Terminal Extension Unique to the Long Isoform of the Shelterin Component TIN2 Enhances Its Interaction with TRF2 in a Phosphorylation- and Dyskeratosis Congenita Cluster-Dependent Fashion

## Supplemental material

- Supplemental file 1 -

  Fig. S1 (CK2 consensus motif conservation), S2 (Telomeres in cells overexpressing TIN2L), S3 (Loss of TIN2L phosphorylation and progressive telomere elongation), S4 (TIN2S, TIN2L, and mutant overexpression and HT1080 population doubling time), S5 (CRISPR/Cas9 targeting of TIN2L expression), S6 (Loss of TIN2L impact on growth), and S7 (Telomere lengths of TIN2L mutant lines)

  PDF, 6.8M
