# Supplementary material for: The C-Terminal Extension Unique to the Long Isoform of the Shelterin Component TIN2 Enhances Its Interaction with TRF2 in a Phosphorylation- and Dyskeratosis Congenita Cluster-Dependent Fashion
Source: Mol Cell Biol. 2018 May 29;38(12):e00025-18. doi: 10.1128/MCB.00025-18 (PMC5974431; doi:10.1128/MCB.00025-18)
Supplement: Supplemental material [file MCB.00025-18_zmb999101768s1.pdf]

```
Q9BSI4     TINF2_HUMAN      370    PPRARK---PVCPPSLCSSVITIGDLVLDSDE--EENGQGEGK-----------------        407
G2HJU8     G2HJU8_PANTR     372    PPRARK---PVCPPSLCSSVITIGDLVLDSDE--EENGQGEGK-----------------        409
G1RZI8     G1RZI8_NOMLE     370    PPRARK---PVCPPSLCSSVITIGDLVLDSDE--EENGQGEGK-----------------        407
F7GSQ3     F7GSQ3_CALJA     370    PPRARK---PVCTPSLCSNVITIGDLVLDSDE--EENGQGEGK-----------------        407
A0A0D9RTU0 A0A0D9RTU0_CHLSB 370    PPRAKK---PVCPPSLCSSIITIGDLVLDSDE--EENGQGEGK-----------------        407
A0A096NXA6 A0A096NXA6_PAPAN 369    PPRARK---PVCPPSLCSSIITIGDLVLDSDE--EEKWPGGRK-----------------        406
F6ZRV3     F6ZRV3_HORSE     372    PPRARK---PVGPPSMCSSVITIGDLVLDSDE--EENGQREGR-----------------        409
M3W4C5     M3W4C5_FELCA     372    PPKARK---PVCPPSLCSSVITIGDLVLDSDE--EENDQREGR-----------------        409
I3M084     I3M084_ICTTR     368    PPRAKN---PVCSPSLCSSVITIGDLVLDSDE--EENSQREGK-----------------        405
F1SGN9     F1SGN9_PIG       371    PPRARK---PACPPSLCSSDITIGDLVLDSEEEEEENGPREGK-----------------        410
M3Y318     M3Y318_MUSPF     371    PPRTRK---PLCPPSLCSSVITIGDLVLDSDE--EENGQKEGR-----------------        408
L8I0G5     L8I0G5_9CETA     372    PPRKS-----VCPPSLYSSDITIGDLVLDSDE--EEDGQRERR-----------------        407
A3KN17     A3KN17_BOVIN     372    PPRKS-----VCPPSLYSSDITIGDLVLDSDE--EEDGQRERR-----------------        407
A0A1U7U6L7 A0A1U7U6L7_TARSY 376    PPRARK---PVCPPSLCSSSITIGDLVLDSDE--EENGQREGKVSRKEQKARKGDGQNKT        430
F1Q2J9     F1Q2J9_CANLF     385    PPRARK---PVCPPSLCSSVITIGDLVLDSDE--EENVQKEGR-----------------        422
W5QCX6     W5QCX6_SHEEP     372    PPRKSAFLPTVCPPSLYSSDITIGDLVLDSDE--EEDGQREGR-----------------        412
K9J163     K9J163_DESRO     371    SPRARK---PVCPPSVCSPVITIGDLVLDSDE--EENGQREGM-----------------        408
G1MCN1     G1MCN1_AILME     372    PPKARK---PLCPPSLCSSVITIGDLVLDSDE--EENGQKEGR-----------------        409
L5M199     L5M199_MYODS     371    PPRARK---PVCPPSLCSPVITIGDLVLDSDE--EENGQRGGM-----------------        408
G5B284     G5B284_HETGA     368    PPKAKK---PACSPFLTTSVITIGDLVLDSEE--EENSQREGK-----------------        405
G1SIM2     G1SIM2_RABIT     366    PPRARK---PVCPPSVCSSVITIGDLVLDSDE--EENSQKDRQ-----------------        403
G3TC22     G3TC22_LOXAF     368    PPKTRK---PASSASLCSPAITIGDLVLDSDD--EDNGQREGK-----------------        405
A0A091DIW8 A0A091DIW8_FUKDA 355    PPKAKK---PACSPFLTTSVITIGDLVLDSEE--EENSQREGK-----------------        392
A0A1S3FW91 A0A1S3FW91_DIPOR 394    PPRAKK---RVCSPSLCSSVITIGDLVLDSDE--EGNSQREGK-----------------        431
H0XYH6     H0XYH6_OTOGA     335    PPRARK---PVSPSSLCSSVITIGDLVLDSDE--EENGQRKGK-----------------        372
A0A1S3WT25 A0A1S3WT25_ERIEU 367    PSRARI---PECPSPMCSSVVTIGDLVLDSEEEESSQ----GK-----------------        402
A0A1U7RC63 A0A1U7RC63_MESAU 364    PPRAKK---PVQSPSLGSSVITIGDLVLDSDE--EENQ-KEGE-----------------        400
L5KHQ5     L5KHQ5_PTEAL     311    PPRARK---PVCLPSLCSPVVTIGDLVLDSDE--EENGQREGR-----------------        348
G3HDD2     G3HDD2_CRIGR     363    PPGAKK---PMQSPSLCSSVITIGDLVLDSDE--EENS-QKEG-----------------        399
Q5XIB8     Q5XIB8_RAT       337    PSRVKK---PVLSPSLCSSVITIGDLVLDSDE--EENNQKEGK-----------------        374
Q8K1K3     Q8K1K3_MOUSE     354    PLTVKK---PVLSPTPCSSVITIGDLVLDSDE--EENNQKEGK-----------------        391
Q8CH11     Q8CH11_MUSSP     351    PLRVKK---PVLSPTPCSSVITIGDLVLDSDE--EENNQKEGK-----------------        388
G3VNG5     G3VNG5_SARHA     377    AVGARE---PVSSPSLCSPVATIGDLVLDSDD--EGNGQRGGK-----------------        414
F6VXG0     F6VXG0_MONDO     377    AVGARE---PVHSPSLCSPVVTIGDLVLDSDD--EGNGQRGGK-----------------        414
                                                    :   *********::   . .
```

>sp|Q9BSI4|TINF2_HUMAN TERF1-interacting nuclear factor 2 OS=Homo sapiens
>tr|G2HJU8|G2HJU8_PANTR TERF1-interacting nuclear factor 2 OS=Pan troglodytes
>tr|G1RZI8|G1RZI8_NOMLE TERF1 interacting nuclear factor 2 OS=Nomascus leucogenys
>tr|F7GSQ3|F7GSQ3_CALJA TERF1 interacting nuclear factor 2 OS=Callithrix jacchus
>tr|A0A0D9RTU0|A0A0D9RTU0_CHLSB TERF1 interacting nuclear factor 2 OS=Chlorocebus sabaeus
>tr|A0A096NXA6|A0A096NXA6_PAPAN TERF1 interacting nuclear factor 2 OS=Papio anubis
>tr|F6ZRV3|F6ZRV3_HORSE TERF1 interacting nuclear factor 2 OS=Equus caballus
>tr|M3W4C5|M3W4C5_FELCA TERF1 interacting nuclear factor 2 OS=Felis catus
>tr|I3M084|I3M084_ICTTR TERF1 interacting nuclear factor 2 OS=Ictidomys tridecemlineatus
>tr|F1SGN9|F1SGN9_PIG TERF1 interacting nuclear factor 2 OS=Sus scrofa
>tr|M3Y318|M3Y318_MUSPF TERF1 interacting nuclear factor 2 OS=Mustela putorius furo
>tr|L8I0G5|L8I0G5_9CETA TERF1-interacting nuclear factor 2 OS=Bos mutus
>tr|A3KN17|A3KN17_BOVIN TERF1 interacting nuclear factor 2 OS=Bos taurus
>tr|A0A1U7U6L7|A0A1U7U6L7_TARSY TERF1-interacting nuclear factor 2 isoform X1 OS=Tarsius syrichta
>tr|F1Q2J9|F1Q2J9_CANLF TERF1 interacting nuclear factor 2 OS=Canis lupus familiaris
>tr|W5QCX6|W5QCX6_SHEEP TERF1 interacting nuclear factor 2 OS=Ovis aries
>tr|K9J163|K9J163_DESRO Putative terf1-interacting nuclear factor 2 OS=Desmodus rotundus
>tr|G1MCN1|G1MCN1_AILME TERF1 interacting nuclear factor 2 OS=Ailuropoda melanoleuca
>tr|L5M199|L5M199_MYODS TERF1-interacting nuclear factor 2 OS=Myotis davidii
>tr|G5B284|G5B284_HETGA TERF1-interacting nuclear factor 2 OS=Heterocephalus glaber
>tr|G1SIM2|G1SIM2_RABIT TERF1 interacting nuclear factor 2 OS=Oryctolagus cuniculus
>tr|G3TC22|G3TC22_LOXAF TERF1 interacting nuclear factor 2 OS=Loxodonta africana
>tr|A0A091DIW8|A0A091DIW8_FUKDA TERF1-interacting nuclear factor 2 OS=Fukomys damarensis
>tr|A0A1S3FW91|A0A1S3FW91_DIPOR TERF1-interacting nuclear factor 2 isoform X1 OS=Dipodomys ordii
>tr|H0XYH6|H0XYH6_OTOGA TERF1 interacting nuclear factor 2 OS=Otolemur garnettii
>tr|A0A1S3WT25|A0A1S3WT25_ERIEU TERF1-interacting nuclear factor 2 isoform X2 OS=Erinaceus europaeus
>tr|A0A1U7RC63|A0A1U7RC63_MESAU TERF1-interacting nuclear factor 2 isoform X1 OS=Mesocricetus auratus
>tr|L5KHQ5|L5KHQ5_PTEAL TERF1-interacting nuclear factor 2 OS=Pteropus alecto
>tr|G3HDD2|G3HDD2_CRIGR TERF1-interacting nuclear factor 2 OS=Cricetulus griseus
>tr|Q5XIB8|Q5XIB8_RAT TERF1 (TRF1)-interacting nuclear factor 2 OS=Rattus norvegicus
>tr|Q8K1K3|Q8K1K3_MOUSE TERF1-interacting nuclear factor 2 OS=Mus musculus
>tr|Q8CH11|Q8CH11_MUSSP TRF1-interacting protein TIN2 OS=Mus spretus
>tr|G3VNG5|G3VNG5_SARHA TERF1 interacting nuclear factor 2 OS=Sarcophilus harrisii
>tr|F6VXG0|F6VXG0_MONDO TERF1 interacting nuclear factor 2 OS=Monodelphis domestica

Figure S1. The CK2 consensus motif is conserved across mammalian TIN2 homologs. Alignment generated using Clustal Omega. Identity of species are shown below.

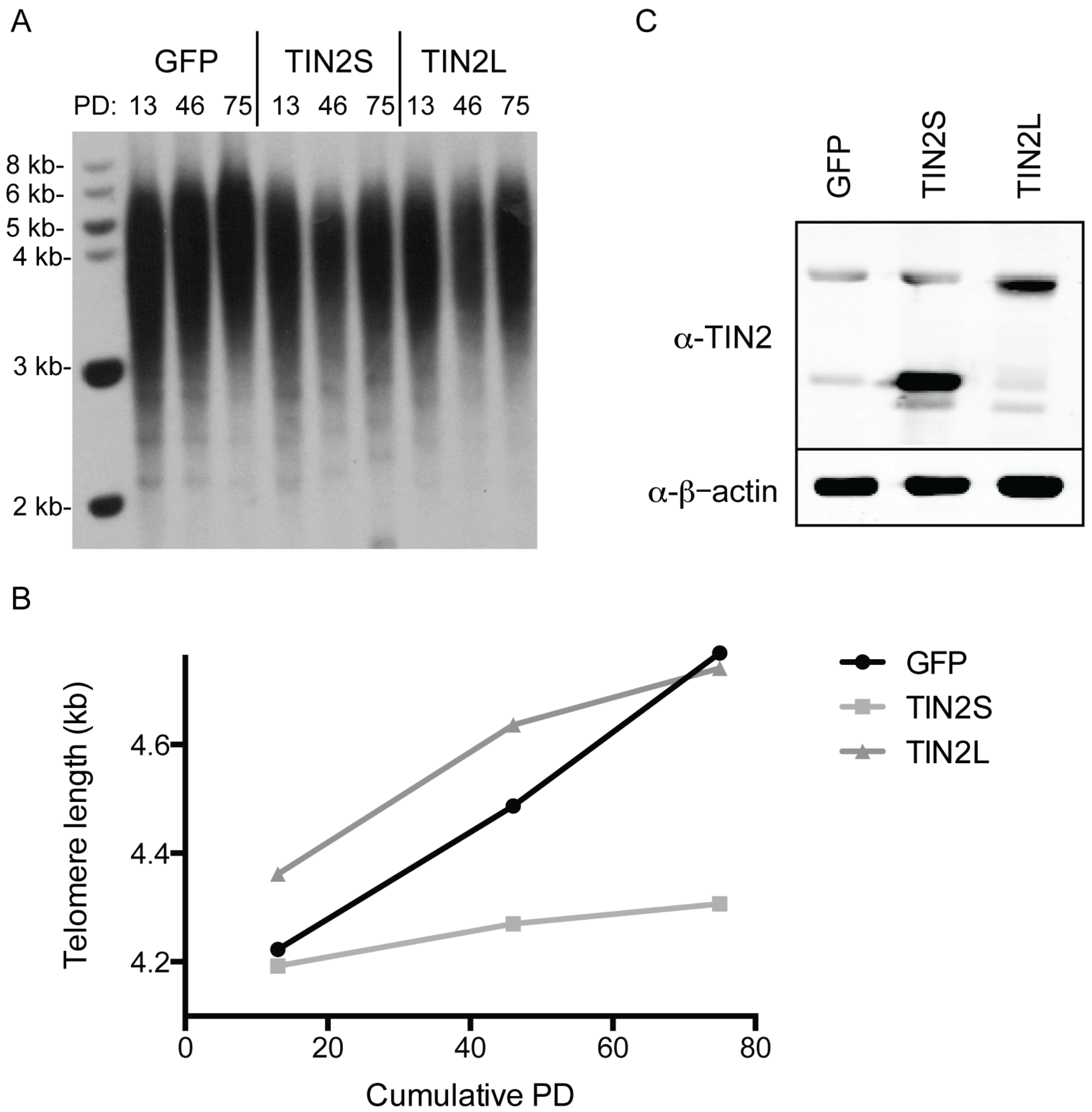


Figure S2. Telomeres in cells overexpressing TIN2L progressively elongated while telomere length remained static in cells overexpressing TIN2S. These cell lines were generated and assayed at a different time from those detailed in Fig. 7. (A) Representative telomere Southern blot analysis of telomere length in kilobasepairs (kb) over time in HT1080 cells overexpressing GFP, TIN2S, or TIN2L. HT1080 cells overexpressing the indicated TIN2 proteins were collected at various times following induction with lentivirus and blasticidin selection and analyzed by the terminal restriction fragment assay. (B) Quantification of (A) using densitometry analysis. (C) Western blot showing TIN2 expression levels in each cell line. Total cell protein lysates were prepared using 2X Laemmli buffer.

Nelson Figure S3

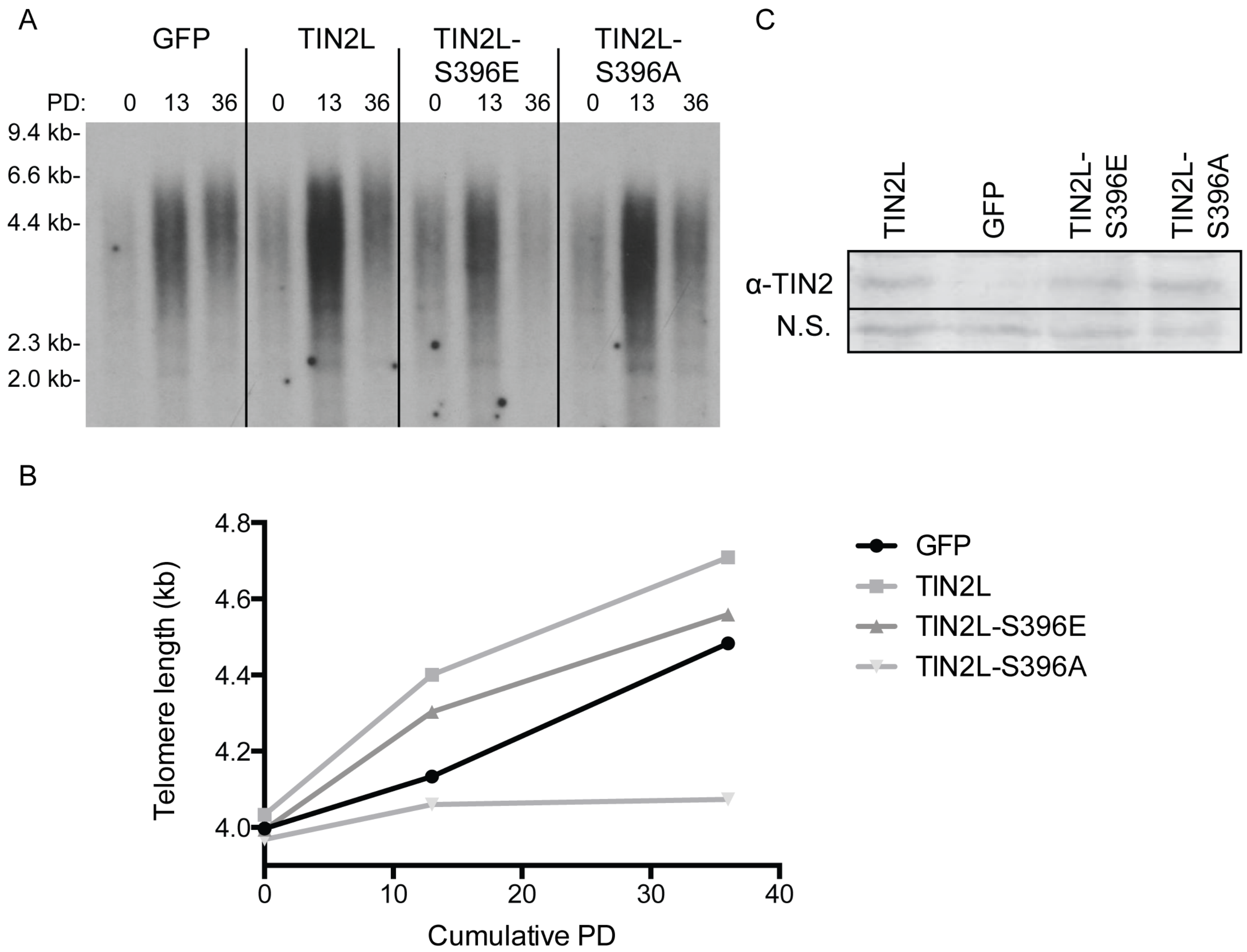


Figure S3. Loss of TIN2L phosphorylation inhibits progressive telomere elongation in telomerase positive HT1080 cells. These cell lines were generated and assayed at a different time from those detailed in Fig. 7. (A) Representative telomere Southern blot analysis of telomere length in kb over time in HT1080 cells overexpressing GFP, TIN2L, TIN2L-S396E, or TIN2L-S396A. HT1080 cells overexpressing the indicated TIN2 proteins were collected at various times following induction with lentivirus and blasticidin selection and analyzed by the terminal restriction fragment assay. (B) Quantification of a using densitometry analysis. (C) Western blot showing TIN2 expression levels in each cell line. Total cell protein lysates were prepared using 2X Laemmli buffer.

Nelson Figure S4

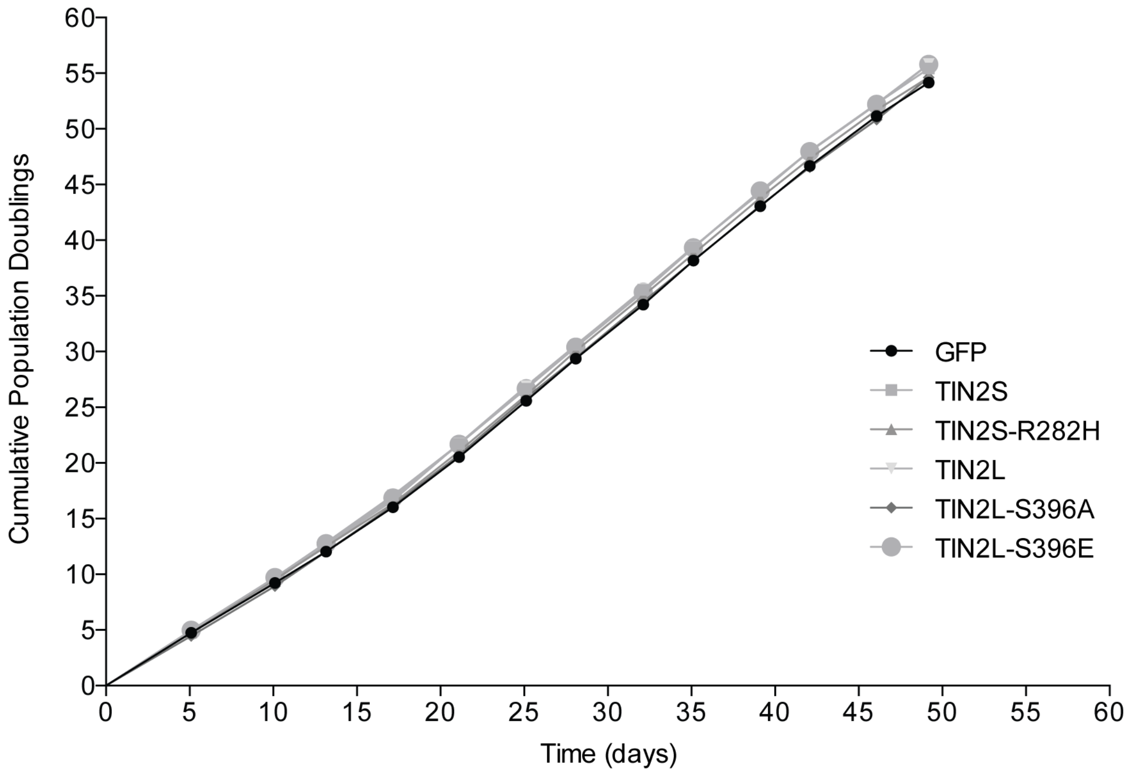


Figure S4. Overexpression of TIN2S, TIN2S-R282H, TIN2L, TIN2L-S396A, and TIN2L-S396E had no effect on HT1080 population doubling time.

**A**

| *TINF2* exon | Name/plasmid | Guide sequence | Quality Score | BLAST results |
|---|---|---|---|---|
| 7 | TINF2ex7-1<br>Ap285 | GAGGTAATAATGATAGTCTC | 76 | 15/20 nucleotides match<br>*KCNC2* |
| 8 | TINF2ex8-1<br>Ap286 | GTCTAAAACCAAGTCCCCTA | 78 | 18/20 nucleotides match<br>*MED1* |

**B**

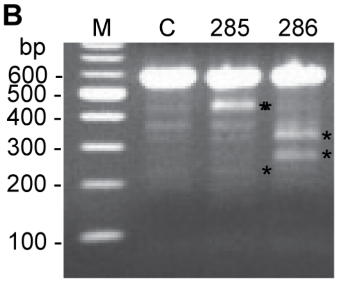


**C**

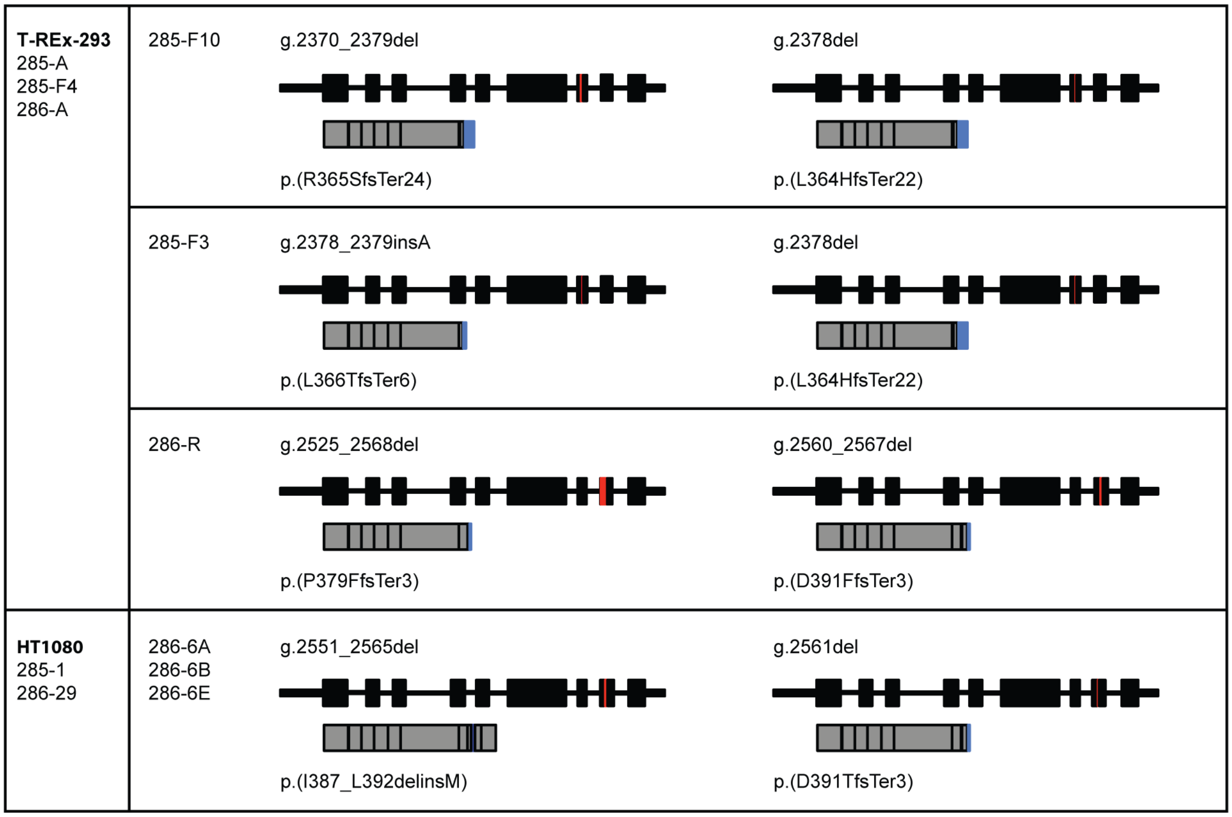


**Figure S5.** CRISPR/Cas9 targeting of TIN2L expression. A. Guides utilized. Quality scores determined using the CRISPR Design Tool. B. Efficiency of site specific targeting as determined using the Guide-It Mutation Detection Kit (Clontech). Predicted fragments indicated by asterisks. C. Mutants generated with genomic changes on each allele as determined by Sanger sequencing of amplicons encompassing the targeted region and corresponding protein changes. Altered sequences indicated in the schematics as colored bars. Individual wild type and mutant clone IDs indicated. 286-6A, 6B, and 6E are individual subclones.

Nelson Figure S6

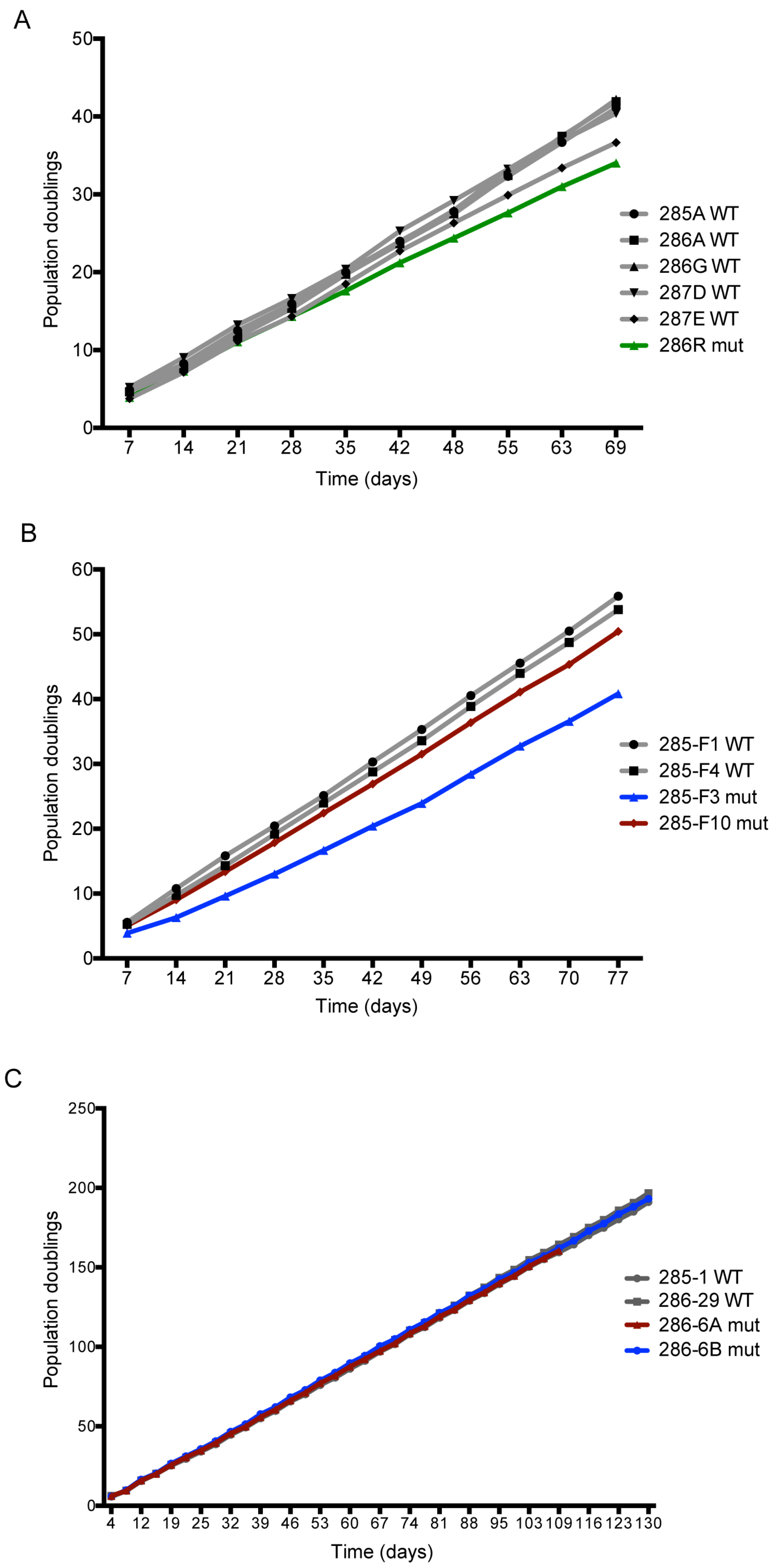


Figure S6. Loss of TIN2L had variable impact on growth. (A) and (B) HT1080 WT and mutant cell lines. (C) T-REx 293 WT and mutant cell lines.

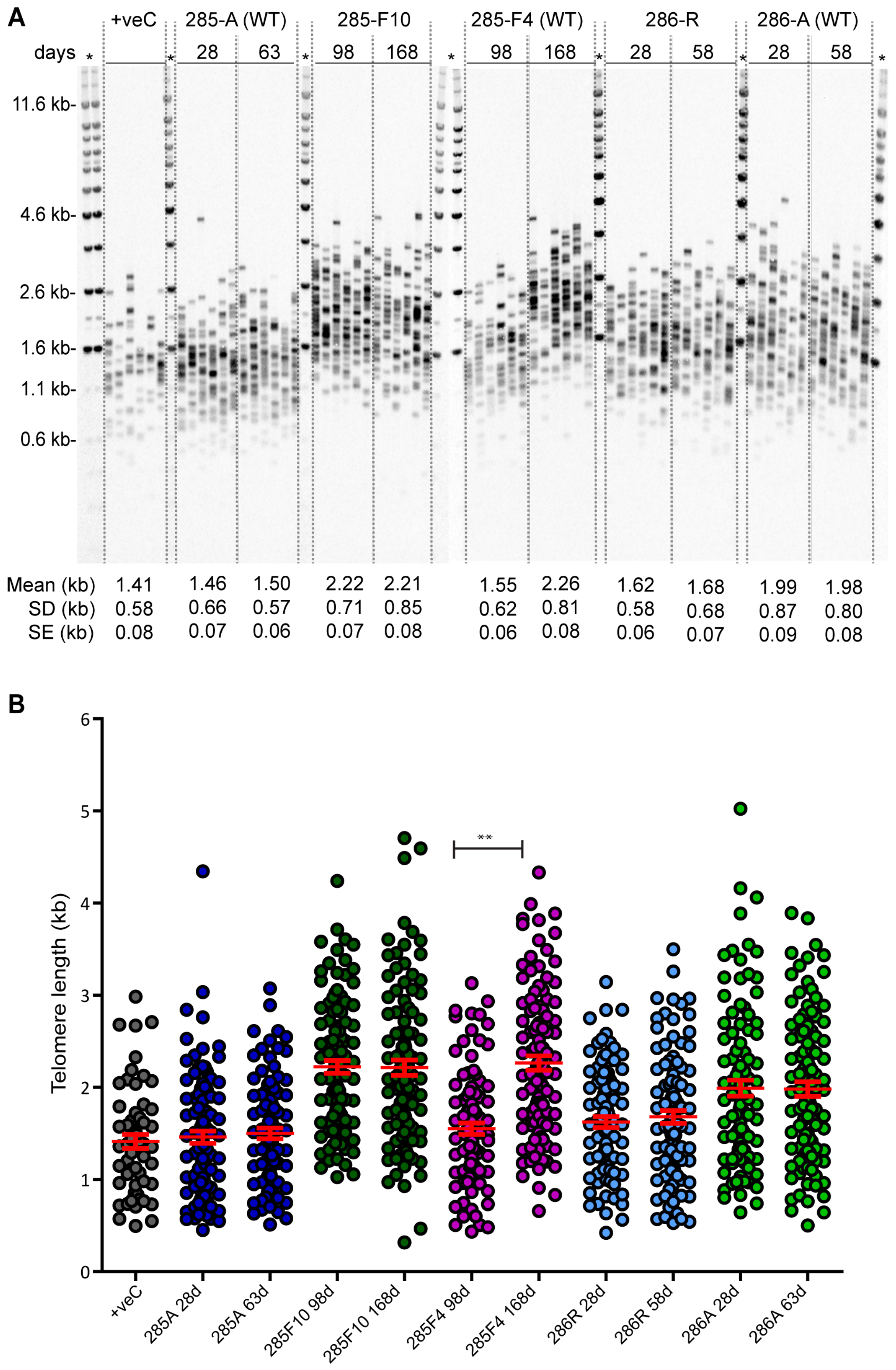


Figure S7. Telomere lengths of TIN2L mutant Flp-In T-REx lines are indistiguishable from TIN2L wildtype lines. (A) 17p STELA analysis of DNA isolated at the indicated numbers of days from the point of clonal line derivation. Mean lengths, standard deviation (SD), and standard error (SE) in kb are indicated. (B) Individual telomere lengths in the designated lines at the designated days.
